# Supplementary material for: Immigrating and vicinity are not risk factors in the prevalence and transmission rate of human T-lymphotropic virus type 1: A Survey in an endemic region of Iran and Afghan refugees
Source: PLOS Glob Public Health. 2023 Jan 10;3(1):e0001484. doi: 10.1371/journal.pgph.0001484 (PMC10021442; doi:10.1371/journal.pgph.0001484)
Supplement: Attachment — Submitted filename: Response to reviewers.docx [file pgph.0001484.s001.docx]

Dear Professor **Catherine Kyobutungi/**Dear Professor **Madhukar Pai**

Editors-in-Chief

***PLOS Global Public Health***

29 October 2022

**Manuscript Reference No. PGPH-D-22-01446**

**Title: “*Immigrating and vicinity are not risk factors in the prevalence and transmission rate of HTLV-1: a survey in an endemic region of Iran and Afghan refugees*”**

We are grateful for the opportunity that has been given to us to revise the manuscript. Thank you so much for your comments, as well as the outstanding suggestions and comments made by the reviewers. The manuscript was critically revised according to the reviewers' comments and suggestions to achieve these purposes. Please find a revised version of the manuscript **PGPH-D-22-01446_R1**, which we would like to resubmit for publication as a research article in ***PLOS Global Public Health***. Your comments and those of the reviewers were highly insightful and enabled us to improve our manuscript's quality significantly. In the following pages are our point-by-point responses to each reviewer's comments and your comments. Revisions in the text are shown using the yellow highlight for additions and strikethrough font for deletions. We hope that this profound revision in the manuscript and our accompanying responses will be sufficient to make our manuscript suitable for publication in ***PLOS Global Public Health***. We appreciate your kind consideration in advance and look forward to hearing from you at your earliest convenience.

With sincere regards,

**Seyed Abdolrahim Rezaee, Ph.D.**

Associate Professor in Immunovirology

Immunology Research Center, Inflammation and Inflammatory Diseases Division

Faculty of Medicine, Mashhad University of Medical Sciences

Azadi-Square, Medical Campus, ZIP code: 9177948564, Mashhad, Iran.

**Tel.:** +98 513 800 2377

**Fax:** +98 513 882 8560

**Cell phone:** +98 915 049 8626

**E-mail:** [RezaeeR@mums.ac.ir](mailto:RezaeeR@mums.ac.ir)

**ORCID:** https://orcid.org/0000-0001-6814-5992

**Responses to the comments of Reviewer # 1**

**[The manuscript entitle: Immigrating and vicinity are not risk factors in the prevalence and transmission rate of HTLV-1: a survey in an endemic region of Iran and Afghan refugees, describe the prevalence of HTLV infection between Afghan refugees in Iran. The authors conclude that HTLV-1 is not endemic in Afghanistan, a war-stricken region with refugees distributed worldwide and the land Silk Road has not been the route of HTLV-1 transmission to Northeastern Iran.**

**It is an important and relevant theme, since we had a lack about HTLV prevalence in Persian region. The methods, results, discussion and conclusion are very clearly, and the English language is adequate.]**

**Response: *Thank you so much for your positive feedback, and valuable time in improving and fortifying this manuscript.***

**Responses to the comments of Reviewer # 2**

***Q1*-[The authors should rename the virus to Huma T-lymphotropic virus. Additionally, the authors stated that all samples from the two refugee camps were negative for HTLV-1 antibody, but refer that anti-HTLV-1 antibodies were detected in serum samples of five cases in the Fariman city group. Please, clarify this point. Where these subjects come from?.]**

**Response Q1: *Thank you so much for your point, it was more cleared in the Methods and Results sections accordingly. Thank you very much for the comments and insightful suggestions which help us to improve the manuscript.***
